# Complete Resection of Cardiac Metastasis Myxoid Liposarcoma

**DOI:** 10.70352/scrj.cr.25-0138

**Published:** 2025-05-09

**Authors:** Kazuhiro Yamazaki, Akio Sakamoto, Takumasa Kosugi, Jiro Sakai, Shuichi Matsuda, Kenji Minatoya

**Affiliations:** 1 Division of Cardiovascular Surgery, Department of Surgery, Shimane University Faculty of Medicine, Izumo, Shimane, Japan; 2 Department of Cardiovascular Surgery, Graduate School of Medicine, Kyoto University, Kyoto, Kyoto, Japan; 3 Department of Orthopaedic Surgery, Graduate School of Medicine, Kyoto University, Kyoto, Kyoto, Japan

**Keywords:** myxoid liposarcoma, cardiac metastasis, ventricular septal rupture, double-patch technique

## Abstract

**INTRODUCTION:**

Myxoid liposarcoma rarely metastasizes to the heart. Therefore, diagnosing cardiac metastases of myxoid liposarcoma is challenging, and treatment is often ineffective. Here, we report a case of cardiac metastasis from a myxoid liposarcoma detected using whole-body MRI, which was successfully treated with a multidisciplinary approach, including surgery, resulting in favorable outcomes.

**CASE PRESENTATION:**

A 61-year-old woman had a history of pelvic myxoid liposarcoma, which was diagnosed and surgically resected at the age of 47. During follow-up, cardiac metastases were identified using whole-body MRI. The patient underwent tumor resection involving the free wall of the left ventricular apex using a cardiopulmonary bypass. The tumor had invaded the ventricular septum, creating a left-to-right shunt due to a fissure in the tumor. This necessitated a double-patch reconstruction following tumor resection. Postoperative radiation therapy was administered as adjuvant treatment. Five years after treatment, there has been no recurrence of the myocardial myxoid-type liposarcoma.

**CONCLUSIONS:**

Complete resection of cardiac metastatic lesions from myxoid liposarcoma was achieved, resulting in favorable outcomes. Early detection of localized cardiac metastases using MRI may enable aggressive surgical interventions.

## INTRODUCTION

Myxoid liposarcoma is a liposarcoma subtype that primarily occurs in adolescents. Unlike most soft-tissue sarcomas, which commonly metastasize to the lungs, myxoid liposarcomas tend to metastasize to soft tissues and bones. Therefore, cardiac metastasis of myxoid liposarcoma is rare. Whole-body MRI is recommended for detecting extrapulmonary metastases of myxoid liposarcoma. We report a case of a patient with cardiac metastasis from myxoid liposarcoma, detected early through whole-body MRI and successfully treated.

## CASE PRESENTATION

A 61-year-old asymptomatic woman was diagnosed with pelvic myxoid liposarcoma at the age of 47. The patient underwent preoperative chemotherapy and extensive tumor resection. The resected specimen did not show any round cell components. Over the following years, the patient underwent surgeries for recurrent tumors in the left gluteal region and metastatic tumors in the left thigh and perineal subcutaneous tissue at the ages of 50, 51, and 54.

**Fig. 1:**
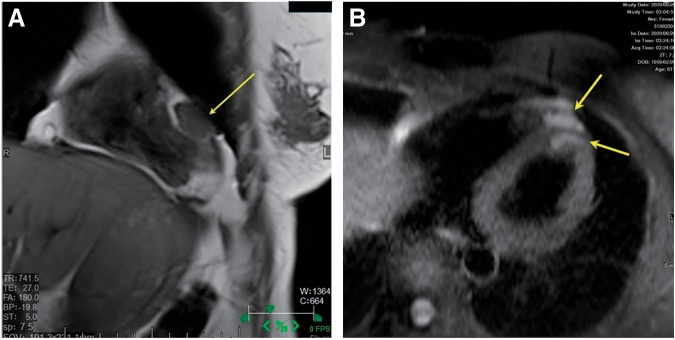
Cardiac MRI images clearly detected the cardiac metastatic lesion with intermediate intensity on T1-weighted images (**A**) and high intensity on T2-weighted fat-suppression images (**B**) (cardiac tumor: yellow arrows).

**Fig. 2:**
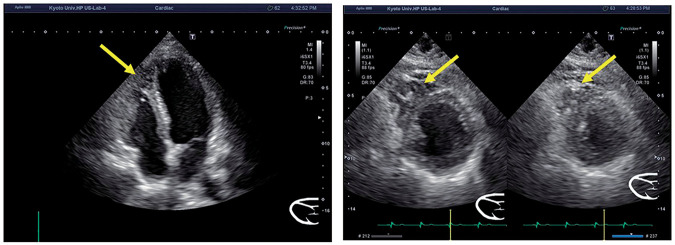
Echocardiography revealed an occupying lesion with a cystic appearance at the apex and within the ventricular septum (cardiac tumor: yellow arrows).

At the age of 61, whole-body MRI revealed a cardiac lesion with high signal intensity on T2-weighted fat-suppression imaging. A retrospective review of the MRI scan taken 6 months earlier confirmed the presence of the lesion. Cardiac MRI was performed to further evaluate the lesion, diagnosing metastatic tumors in the left ventricular epicardium and myocardium. Tumor resection was planned (**Fig. 1**). Echocardiography revealed an occupying lesion with a cystic appearance at the apex and within the ventricular septum (**Fig. 2**).

The surgery was approached via median sternotomy. The pericardium was opened, revealing a small amount of yellow, clear pericardial fluid without hemorrhagic effusion. The tumor adhered to, but did not invade, the pericardium, protruding from the epicardium. It measured approximately 3 cm in diameter and 5 mm in height on the surface, appearing as a disc-shaped, flat, white mass with clear margins. An anterior descending artery branch ran over the tumor.

**Fig. 3:**
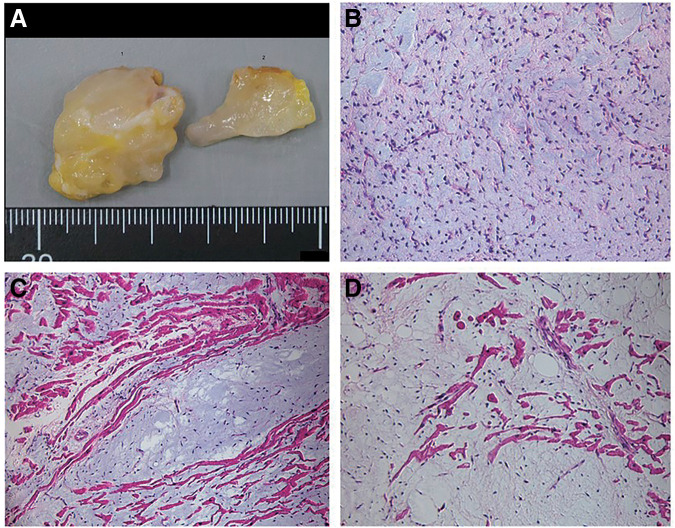
Resected cardiac tumor (**A**). The histologic evaluation showed a proliferation of bland stellate tumor cells embedded in a myxoid matrix with a prominent plexiform capillary network (**B**). The tumor tissue invaded into the myocardium (**C** and **D**).

Cardiopulmonary bypass was initiated, and tumor resection was performed under cardioplegic arrest. The tumor was carefully dissected from the left anterior descending artery, which had 2 branches infiltrating the tumor. These branches were clipped and divided. As the dissection continued along the tumor margins, most of the myocardium was easily separated, because the tumor was white, elastic, and firm, did not invade the surrounding tissue, and had well-defined borders. However, at 2 or 3 locations, mainly in the center of the tumor, the tumor continuously extended deep into the interventricular septum. After completing dissection in these areas (**Fig. 3A**), the surface portion of the tumor was quickly submitted for intraoperative pathological examination, which confirmed the diagnosis of myxoid liposarcoma.

**Fig. 4:**
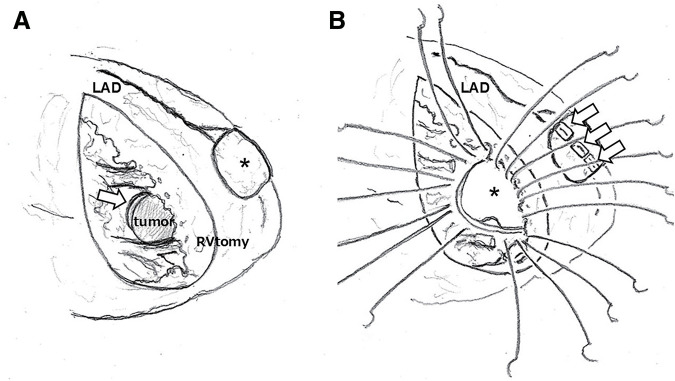
Operating schemes. (**A**) Attached the pericardial patch (black asterisk) using glue after resecting the visible tumor and cryoablation of the affected area. The tumor in the ventricular septum was identified immediately below the apex, and a slit at the superior end of the tumor is shown (arrow). (**B**) Suture of the penetrating wall with 3 stitches using 3-0 monofilament sutures with felt pledget (arrows). Reconstruction using 2 bovine pericardial patches, 1 for the left ventricle (asterisk) and 1 for the right ventricle, using the double-patch technique. LAD, left anterior descending artery; RVtomy, right ventriculotomy

We successfully resected most of the visible tumor in the myocardium of the left ventricular free wall. Although a small portion of tumor was found to have invaded the ventricular septum, complete surgical resection was deemed technically challenging and risky. Therefore, we opted for the cryoablation of affected area and BioGlue Surgical Adhesive (CryoLife, Kennesaw, GA, USA) to affix the ablated surface with an autologous pericardial patch for reinforcement. The aorta was declamped, and spontaneous beating resumed naturally. However, transesophageal echocardiography revealed a significant left-to-right shunt flow of the ventricular septum. It was suspected that the tumor in the ventricular septum had become fragile and ruptured or perforated. Due to the large shunt volume, the aorta was clamped again, and an incision was made on the anterior surface of the right ventricle, parallel to the left anterior descending artery. Although the tumor in the ventricular septum was immediately identified, locating the septal perforation proved challenging. To locate the defect, a left ventricular vent cannula was inserted, and blood was injected into the left ventricle through it. This revealed a slit at the superior end of the tumor, confirming that blood was leaking from the margin between the well-defined tumor and the surrounding tissue (**Fig. 4A**). Approximately 4 cm of the ventricular septum centered on the tumor was resected. The area of the cardiac surface with autologous pericardium was sutured by the penetrating wall with 3 stitches using 3-0 monofilament sutures with felt pledget, and the remainder was sutured around the resected ventricular septum with 3-0 monofilament sutures. The septum was reconstructed with 2 bovine pericardial patches—1 for the left ventricle and 1 for the right ventricle—employing the double-patch technique (**Fig. 4B**). The right ventricular incision line was closed in 2 layers with 3-0 monofilament reinforced with felt strips. Weaning from cardiopulmonary bypass was performed with intra-aortic balloon support. The total cardiopulmonary bypass time was 287 min, with the aortic clamping times of 92 and 169 min, for a cumulative total of 261 min.

Histologic examination of the resected specimen revealed a proliferation of bland stellate tumor cells embedded in a myxoid matrix with a prominent plexiform capillary network (**Fig. 3B**). No mitotic activity or pleomorphism was observed. These histologic features were consistent with those of a low-grade myxoid liposarcoma. The neoplastic cells invaded the cardiac muscle fibers (**Fig. 3C** and **3D**).

The patient was admitted to the intensive care unit for 1 month because of postoperative low cardiac output syndrome. Adjuvant radiotherapy (60 Gy in 30 fractions) was administered on postoperative day 77. The patient was discharged on postoperative day 124. Postoperative echocardiography revealed no left-to-right shunt flow through the ventricular septum. The left ventricular ejection fraction was 66.1% preoperatively, declined to 20.6% 3 months after surgery but recovered to 63.9% 2 years later. Postoperative MRI and CT scans showed no recurrence of cardiac myxoid liposarcoma. The patient continues to attend outpatient follow-ups and remains in good health 5 years after surgery. Following echocardiography, CT and MRI scans of the whole body are performed every year, and the myxoid liposarcoma has not recurred, and the patient remains in good health. We plan to continue to follow up with the patient in the future.

## DISCUSSION

Myxoid liposarcoma is characterized by slow-growing lesions of the lower extremities.^1)^ Its 5-year survival rate is 90%, and the 10-year survival rate is 85%. Lesions containing >5% round-cell components are associated with a poor prognosis, with a 5-year survival rate of only 60%.^2)^ Unlike most sarcomas, which predominantly metastasize to the lungs, 45%–78% of myxoid liposarcoma metastases occur in extrapulmonary sites,^3,4)^ including bones and soft tissues. Cardiac metastasis is rare, with approximately 40 reported cases, including those involving the pericardium.^5–7)^

Whole-body MRI has been reported to be useful in identifying extrapulmonary myxoid liposarcoma metastases^1)^; 43% of extrapulmonary myxoid liposarcoma metastases that were not detected by CT were identified by whole-body MRI.^8)^ In this case, MRI facilitated the early detection of asymptomatic cardiac metastasis. The interval between primary treatment and the development of cardiac metastasis varies from 1 to 25 years.^5–7)^ Early detection using whole-body MRI during follow-up can identify unexpected sites of metastasis, including the heart. Patients with myxoid liposarcoma metastatic to the heart are often asymptomatic for long periods of time but may rapidly develop heart failure due to obstruction at the metastatic site.^5–7,9)^ Resection of the cardiac metastases may offer the best chance for prolonged survival and is more successful when performed at an early stage. Therefore, we believe that aggressive surgical resection is effective if the tumor is detected at an early stage. However, since the tumor is a nonnormal tissue that can rupture intraoperatively, it should be handled with great care during surgery.

## CONCLUSIONS

We managed a case of cardiac metastasis of myxoid liposarcoma detected early using whole-body MRI. Complete surgical resection followed by postoperative radiotherapy yielded favorable outcomes. Therefore, cardiac metastases of myxoid liposarcoma warrant aggressive surgical intervention when detected early and confined to localized areas.

## ACKNOWLEDGMENTS

None.

## DECLARATIONS

### Funding

None of the authors received any funding for this study.

### Authors’ contributions

The authors provided clinical care for the patients.

Drs. Akio Sakamoto, Takumasa Kosugi, and Shuichi Matsuda provided advice on postoperative adjuvant therapy.

The authors read and approved the final manuscript.

### Availability of data and materials

Data sharing is not applicable to this article, as no datasets were generated or analyzed during the study.

### Ethics approval and consent to participate

This work does not require ethical considerations or approval. The patient signed informed consent to participate in our study.

### Consent for publication

Informed consent was obtained from the patient for the publication of this case report and any accompanying images.

### Competing interests

The authors declare that they have no competing interests.
